# Associations Between the Use of Renin–Angiotensin System Inhibitors and the Risks of Severe COVID-19 and Mortality in COVID-19 Patients With Hypertension: A Meta-Analysis of Observational Studies

**DOI:** 10.3389/fcvm.2021.609857

**Published:** 2021-04-26

**Authors:** Xiao-Ce Dai, Zhuo-Yu An, Zi-Yang Wang, Zi-Zhen Wang, Yi-Ren Wang

**Affiliations:** ^1^Department of Cardiology, Affiliated Hospital of Jiaxing University, Zhejiang, China; ^2^Peking University People's Hospital, Beijing, China; ^3^China-Japan Friendship Hospital, Beijing, China

**Keywords:** angiotensin receptor blockers, angiotensin converting enzyme inhibitors, coronavirus disease 2019, hypertension, death

## Abstract

Angiotensin-converting enzyme inhibitors (ACEIs) and angiotensin receptor blockers (ARBs) share a target receptor with severe acute respiratory syndrome coronavirus 2 (SARS-CoV-2). The use of ACEIs/ARBs may cause angiotensin-converting enzyme 2 receptor upregulation, facilitating the entry of SARS-CoV-2 into host cells. There is concern that the use of ACEIs/ARBs could increase the risks of severe COVID-19 and mortality. The impact of discontinuing these drugs in patients with COVID-19 remains uncertain. We aimed to assess the association between the use of ACEIs/ARBs and the risks of mortality and severe disease in patients with COVID-19. A systematic search was performed in PubMed, EMBASE, Cochrane Library, and MedRxiv.org from December 1, 2019, to June 20, 2020. We also identified additional citations by manually searching the reference lists of eligible articles. Forty-two observational studies including 63,893 participants were included. We found that the use of ACEIs/ARBs was not significantly associated with a reduction in the relative risk of all-cause mortality [odds ratio (OR) = 0.87, 95% confidence interval (95% CI) = 0.75–1.00; *I*^2^ = 57%, *p* = 0.05]. We found no significant reduction in the risk of severe disease in the ACEI subgroup (OR = 0.95, 95% CI = 0.88–1.02, *I*^2^ = 50%, *p* = 0.18), the ARB subgroup (OR = 1.03, 95% CI = 0.94–1.13, *I*^2^ = 62%, *p* = 0.48), or the ACEI/ARB subgroup (OR = 0.83, 95% CI = 0.65–1.08, *I*^2^ = 67%, *p* = 0.16). Moreover, seven studies showed no significant difference in the duration of hospitalization between the two groups (mean difference = 0.33, 95% CI = −1.75 to 2.40, *p* = 0.76). In conclusion, the use of ACEIs/ARBs appears to not have a significant effect on mortality, disease severity, or duration of hospitalization in COVID-19 patients. On the basis of the findings of this meta-analysis, there is no support for the cessation of treatment with ACEIs or ARBs in patients with COVID-19.

## Introduction

Coronavirus disease 2019 (COVID-19), which is caused by severe acute respiratory syndrome coronavirus 2 (SARS-CoV-2), has initiated a global epidemic. SARS-CoV-2 uses the receptor angiotensin-converting enzyme 2 (ACE2) to gain entry into target cells (1–3). ACE2 is part of the renin–angiotensin system (RAS). Because RAS inhibitors, such as angiotensin-converting enzyme inhibitors (ACEIs) and angiotensin receptor blockers (ARBs), increase the levels of ACE2, the protein that facilitates the entry of SARS-CoV-2 into cells, there are concerns that these drugs could increase the risks of severe COVID-19 and mortality (4). Evidence that ACEIs and ARBs might upregulate ACE2 in several organs, including the lungs and heart (5), supported the hypothesis widely reported by the press that their use might increase susceptibility to infection with SARS-CoV-2 and that their discontinuation might therefore be an appropriate preventive measure (6). Based on these facts and observations, the hypothesis has been developed that their use may affect human susceptibility to infection with SARS-CoV-2.

However, in animal models, ACEIs and ARBs are protective against acute lung injury, and pretreatment with ACEIs or ARBs may reduce the extent of experimentally induced lung injury and improve outcomes, an effect mediated by inhibition of the RAS (7).

Activation of the RAS can cause widespread endothelial dysfunction and varying degrees of injury to multiple organs (heart, kidney, and lung) (8). Thus, researchers have hypothesized that ACEIs/ARBs could theoretically be beneficial and reduce the risk of severe disease in patients with COVID-19.

These possibilities pose a dilemma for cardiologists in terms of whether they should recommend discontinuing treatment with ACEIs/ARBs. Therefore, we performed a large-scale meta-analysis to estimate the associations between ACEIs/ARBs use and the risk of severe COVID-19 and prolonged hospitalization due to COVID-19 (9).

## Methods

### Literature Search

The present analysis was conducted in accordance with published PRISMA (Preferred Reporting Items for Systematic Reviews and Meta-Analyses) and MOOSE (Meta-Analysis of Observational Studies in Epidemiology) guidelines (10). The meta-analysis was registered with the International Prospective Register of Systematic Reviews (PROSPERO identifier: CRD42020183921). Electronic searches were conducted in PubMed, EMBASE, Cochrane Library, and MedRxiv.org from December 1, 2019, to June 20, 2020. As of the date the searches were performed, no randomized controlled clinical trials had been published; therefore, only observational studies were included. We also identified additional citations by manually searching the reference lists of eligible studies.

We used the following medical subject headings and keywords to search for articles related to COVID-19: COVID-19, severe acute respiratory syndrome coronavirus 2, 2019-nCoV, and SARS-CoV-2; and the following search terms related to ACEIs/ARBs: renin–angiotensin system, angiotensin-converting enzyme inhibitor, and angiotensin II receptor blockers (Supplementary Table 1).

### Eligibility Criteria

**Figure 1:**
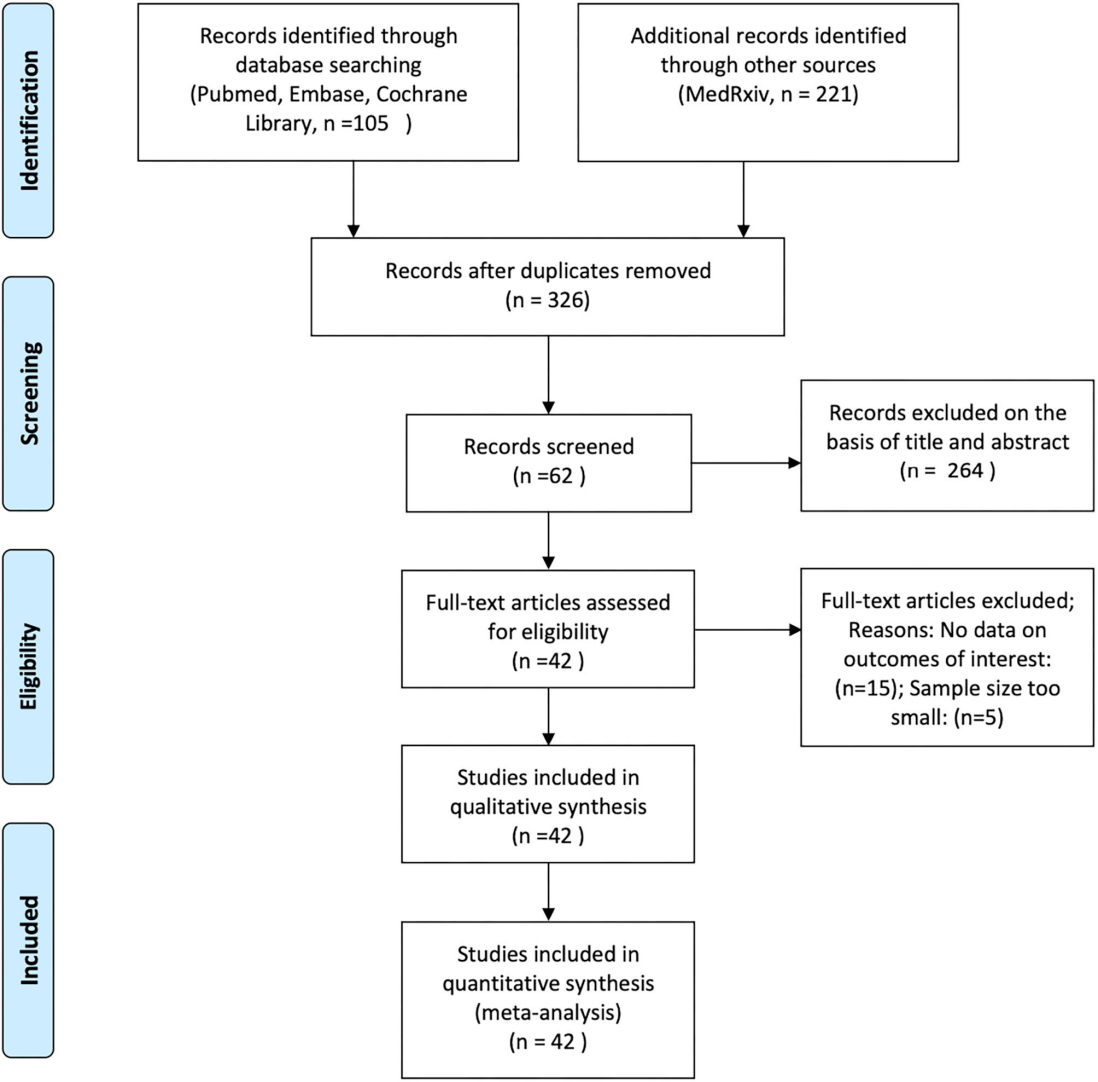
Flowchart of article selection process.

Two of the authors (ZA and ZW) independently analyzed the titles and abstracts of all articles retrieved from these searches to ascertain whether they met the inclusion criteria. We assessed the full texts of the initially eligible articles based on the PICOTS (Population, Intervention, Comparator, Outcome, Timing and Setting) framework, and articles were selected according to the following criteria: (1) articles reporting observational studies, including cohort studies and case-control studies; (2) articles that analyzed the effects of ACEIs/ARBs on COVID-19 in adult patients with hypertension; (3) articles that contained data on mortality, disease severity, and hospitalization durations in COVID-19 patients; and (4) articles that enrolled at least 50 patients (Figure 1).

### Data Extraction

Two investigators (ZA and ZW) independently extracted the relevant data with a predetermined data collection table. Any discrepancies were settled by consensus or consultation with a third investigator (DXC). All the included data were aggregate data, and no patient-level data were available.

### Quality Assessment

Study quality was assessed with the Newcastle–Ottawa Scale (NOS, maximum 9 points), which rates studies based on three parameters: the selection of groups, the comparability, and the ascertainment of outcome and exposure. The NOS can be used to evaluate the overall risk of bias in non-randomized studies.

### Outcomes of Interest

Data for all-cause mortality, severity, and hospitalization duration in COVID patients were collected. Severe cases of COVID-19 were generally characterized by dyspnea, a respiratory rate >30 breaths/min, a blood oxygen saturation level <93% on room air, a Pao_2_/Fio_2_ ratio <300, and/or infiltration of >50% of the lung within 24–48 h, or according to the criteria defined in each included study (11).

### Statistical Analysis

The adjusted odds ratios (ORs) and hazard ratios for all-cause mortality, severe disease, and prolonged hospitalization duration in COVID patients were reported in these studies. Both adjusted and unadjusted ORs were initially considered in the analysis. We pooled the adjusted ORs, which were derived from multivariate analyses. We used the *I*^2^ statistic to assess the heterogeneity of the summary estimates, and a value >50% was considered evidence of significant heterogeneity (12). A random-effects model was used because the *I*^2^ statistic was >50%. To assess publication bias, we constructed a funnel plot and adopted the Begg rank correlation method (*p* < 0.05 indicated significant bias). We used Stata version 14.0 (Stata Corp., College Station, TX, USA) for all calculations. We used RevMan 5.3 (Nordic Cochrane Centre, Cochrane Collaboration) to generate forest plots to show the results for the individual studies and the pooled analysis.

## Results

### Characteristics and Quality of Included Studies

Among the 42 studies included, 14 were performed in Europe, 7 in the United States, 4 in Korea, 1 in Iran, and 16 in China. All studies were published within the past 6 months, and all were observational studies. We confirmed that all observational studies had adequate inclusion and exclusion criteria and an appropriate justification for the selection of the cohort. We collected and sorted the data on intervention measures and examination results obtained from the electronic medical records in all studies.

We summarized the baseline characteristics in each study in Table 1 (8, 13–43, 45–48, 50, 52–56). The identified studies included 63,893 patients with COVID-19. Of these, 20,686 were taking ACEIs/ARBs. Thirty-five studies adjusted their analyses for comorbidities (including diabetes mellitus, cardiovascular disease, and chronic kidney disease). Seven studies did not describe the controls; however, the remaining studies described the controls as patients who had COVID-19 but had not been exposed to ACEIs/ARBs. Seven studies compared the hospitalization durations (days) between the ACEI/ARB and non-ACEI/ARB groups. Twenty-nine studies (69.04%) described mortality in the study populations.

**Table 1:** Baseline characteristics of patients assessed in the studies included in the meta-analysis.

**Study authors, year of publication, location**	**Total**					**ACEI/**	**Characteristics of controls**	**Confounding factors adjusted for in the analyses**	**NOS score (max = 9)**
	**Number**	**Deaths, *n* (%)**	**Age, mean ± SD or (range)**	**Gender (male, %)**	**Comorbidities, *n* (%)**	**ARB, *n***			
Andrea et al. (13), Italy	191	42 (28)	NA	68.6	CAD: 14.7% Heart Failure: 4.7% DM: 14.7% COPD: 5.2% CKD: 26.2%	69	Patients with hypertension and COVID-19 that were taking other anti-hypertension drugs.	Age, Heart failure, CKD.	7
Ashraf et al. (14), Iran	100	12	58 (48–68)	64%	DM: 26% CAD: 19%	19	Patients with hypertension and COVID-19 that were taking other anti-hypertension drugs.	The analyses were not adjusted for multiple comparisons.	6
Baker et al. (15), UK	316	81	75 (60–83)	55%	DM: 27% CAD: 21% CKD: 24%	311	Patients with hypertension and COVID-19 that were taking other anti-hypertension drugs.	The analyses were not adjusted for multiple comparisons.	6
Bean et al. (16), UK	205	53 (25.9%)	63 ± 20	52%	DM: 62 (30.2%) CVD: 30 (14.6%)	46	Patients with hypertension and COVID-19 that were taking other anti-hypertension drugs.	Age, gender, comorbidities (hypertension, DM, IHD, and heart failure)	7
Benelli et al. (17), Italy	411	72	66.8 ± 16.4	87%	DM: 16% CAD: 23%	135	Patients with hypertension and COVID-19 that were taking other anti-hypertension drugs.	Bonferroni correction was used to adjust for multiple testing.	8
Bravi et al. (18), Italy	543	129 (very severe/lethal)	NA	NA	NA	450	Patients with hypertension and COVID-19 that were taking other anti-hypertension drugs.	All estimates have been adjusted for age, gender, diabetes, major cardiovascular diseases, COPD, cancer. and renal diseases.	8
Chen et al. (19), China	123	31	57.7 ± 12.7	43%	DM: 11% CAD: (12%)	11	Patients with hypertension and COVID-19 that were taking other anti-hypertension drugs.	The analyses were not adjusted for multiple comparisons.	7
Choi et al. (20), Korea	1,585	192	66.5 ± 14	42.80%	DM: 44.9% Chronic lung diseases: 19.5%	892	Propensity score-matched hospitalized patients with COVID-19 that were taking other anti-hypertension drugs.	Adjusted for age, sex, region of hospitals, comorbidities (diabetes, chronic lung disease, and major neurologic diseases), Charlson comorbidity index, and treatment modalities.	7
Dauchet et al. (21), France	288	NA	NA	62%	DM: 40 (13.89%) CVD: 48 (16.67%) Pulmonary disease: 31 (10.76%) CKD: 9 (3.13%)	62	NA	Age, gender, weight, comorbidities (DM, pulmonary disease, kidney diseases, CVD)	7
De Spiegeleer et al. (22), Belgium	154	NA	86 ± 7	33%	DM: 18%	30	Residents at two elderly care homes with COVID-19 that were taking other anti-hypertension drugs.	Age, sex, functional status, diabetes mellitus, hypertension	7
Felice et al. (23), Italy	133	33	72.8 ± 12.3	64.70%	DM: 25.6% CAD: 42.1% COPD: 10.5%	82	Hospitalized patients with COVID-19 that were taking other anti-hypertension drugs.	Adjusted for age, gender, body mass index, days with symptoms prior to admission, previous cardiovascular events, diabetes, and cancer.	9
Feng et al. (24), China	476	38	53.0 (40.0–64.0)	56.90%	DM: 49 (10.29%) CVD: 38 (7.98%) Pulmonary disease: 22 (4.62%) CKD: 4 (0.84%)	35	Patients with hypertension hospitalized with COVID-19 that were taking other anti-hypertension drugs matched to the experimental group according to disease severity.	Age, sex, smoking, alcohol consumption	7
Fosbøl et al. (25), Denmark	4,480	478	54.7 (40.9–72.0)	47.90%	DM: 411 Heart Failure: 243 COPD: 634 CKD: 172	895	Hospitalized patients with COVID-19 with hypertension that were taking other anti-hypertension drugs.	Fully adjusted model includes the following covariates: age; sex; highest obtained educational level; medical history of myocardial infarction, heart failure, kidney disease, stroke, peripheral artery disease, atrial fibrillation, diabetes, chronic obstructive pulmonary disease, and malignancy; and use of the following concomitant medications: other antihypertensive drugs, lipid-lowering drugs, and anticoagulation.	8
Gao et al. (26), China	850	34	64.24 (11.2)	52.10%	DM: 26.8% CAD: 16.7%	183	Hospitalized patients with COVID-19 with hypertension that were taking other anti-hypertension drugs.	Adjusted for age, sex, medical history of diabetes, insulin-treated diabetes, myocardial infarction, underwent PCI/CABG, renal failure, stroke, heart failure, and COPD.	9
Giorgi et al. (27), Italy	2,653	217	63.2	50%	DM: 12% CAD: 7%	818	Symptomatic patients with COVID-19 that were taking other anti-hypertension drugs.	Adjused for age and comorbidities.	9
Guo et al. (28), China	187	43	58.5 ± 14.6	49%	DM: 15% CAD: 11.2%	19 (10%)	Patients with COVID-19 symptoms that required hospitalization that were taking other anti-hypertension drugs.	NA	8
Huang et al. (29), China	50	2	61.7 ± 12.9	54%	DM: 8% CAD: 2%	20	Patients with COVID-19 with hypertension that were taking other anti-hypertension drugs.	Unadjusted comparisons	8
Ip et al. (30). USA	1,129	399	NA	NA	NA	460	Patients with COVID-19 with hypertension that were taking other anti-hypertension drugs.	Adjusted for age, the effect of hypertension on mortality was greatly diminished, with a reduction in odds-ratio by over half; and completely disappeared when adjusted for other major covariates.	7
Jung et al. (31), Korea	5,179	84	44.6 ± 18	44%	DM: 17% CAD: 1% CKD: 5%	762	Patients with COVID-19 with hypertension that were taking other anti-hypertension drugs.	Adjusted for age, sex, Charlson Comorbidity Index, immunosuppression, and hospital type.	7
Jurado et al. (32), Spain	290	NA	NA	NA	NA	190	Patients with COVID-19 with hypertension that were not exposed to ACEI or ARB.	NA	7
Khera et al. (33), USA	10,196	1,128	NA	54%	DM: 48% CAD: 5% CKD: 27%	6,040	Patients with COVID-19 with hypertension that were not exposed to ACEI or ARB. Pairwise comparisons from propensity score matched cohorts. In hospital patient and outpatient were compared.	NA	7
Kim et al. (34), USA	2,491	420	62 (50–75)	53%	DM: 33% CAD: 14% CKD: 16%	573	Patients with COVID-19 with hypertension that were not exposed to ACEI or ARB	Adjusting for age group, sex, and race/ethnicity and underlying conditions.	8
Lee et al. (35), Korea	8,266	112	44.4 ± 19.1	38%	DM: 17% CAD: 6%	977	Hospitalized patients with COVID-19 with hypertension that were not exposed to ACEI or ARB	Adjusted for age, sex, the history of comorbidities (hypertension, diabetes mellitus, cancer, COPD, stroke, coronary artery disease, heart failure, and chronic kidney disease) before diagnosis of SARS-CoV-2.	7
Li et al. (36), China	362	77	66.0 (59.0–73.0)	52.20%	DM: 127 (35.1%) CVD: 62 (17.13%) CKD: 35 (9.67%)	118	Patients with hypertension hospitalized with COVID-19 that were taking other anti-hypertension drugs.	Age, gender, comorbidities (DM, cerebrovascular disease, coronary heart disease, digestive disorders, respiratory disease, neurological disease, solid tumor, CKD)	6
Liabeuf et al. (37), France	268	63	73 (61–84)	58%	DM: 21% CAD: 61% COPD: 10% CKD:7% Restrictive lung disease: 6%	96	Hospitalized patients with COVID-19 with hypertension that were not exposed to ACEI or ARB	Adjustment for age, sex, coronary heart disease, BMI.	8
Liu et al. (38), China	78	NA	65.2 ± 10.7	55%	NA	12	Patients with COVID-19 with hypertension that were not exposed to ACEI or ARB.	Adjustment was by multivariable logistic regression modeling with sex variable	7
Mancia et al. (39), Italy	6,272	NA	68 ± 13	63%	CVD: 1,891 (30.1%) CKD: 651 (10.4%)	2,896	30,759 beneficiaries of the Regional Health Service, matched to the experimental group according to sex, age, and municipality of residence	Drugs (antihypertensive drugs, oral antidiabetic drugs), comorbidities (CVD, respiratory disease, kidney disease, cancer), and chronic related conditions	7
Mehta et al. (40), USA	1,735	NA	NA	57%	DM: 46% CAD: 22%	214	Patients with COVID-19 with hypertension that were not exposed to ACEI or ARB.	Unadjusted comparisons	7
Meng et al. (41), China	417	NA	64.50 (55.80–69.00)	57.10%	DM: 5 (11.9%) CVD: 8 (19.0%) Pulmonary disease: 225 (8.5%)	17	Patients with COVID-19 that had hypertension comorbidity, based on treatment, but were taking non-ACEI/ARB anti-hypertension drugs.	Age, sex, symptoms, and signs	8
Peng et al. (42), China	112	17	62 (55–67)	47%	DM: 20% CAD: (55%)	22	Patients with COVID-19 symptoms that required hospitalization that have hypertension taking other anti-hypertension drugs.	NA	6
Rentsch et al. (43), USA	585	17	66.1 (60.4–71)	52%	DM: 30% CAD: 15%	263	Patients with symptoms that required hospitalization with COVID-19 that were taking other anti-hypertension drugs	Age, sex, race/ethnicity, residence type	7
Reynolds et al. (44), USA	5894	NA	NA	NA	NA	1,692	Patients with COVID-19 that had hypertension comorbidity, based on treatment, but were taking non-ACEI/ARB anti-hypertension drugs	Age, sex, race, ethnic group, BMI, smoking history, history of hypertension, myocardial infarction, heart failure, DM, CKD, obstructive lung disease, and other classes of medication	9
Rhee et al. (45), Korea	832	34	NA	53%	DM: 100% CAD: 27% CKD: 19%	327	Patients with COVID-19 that were taking non-ACEI/ARB anti-hypertension drugs	Adjustment for age, sex, comorbidity, and medication	8
Richardson et al. (46), USA	1,366	NA	63 (52–75)	60%	NA	456	Patients with COVID-19 with hypertension that were taking non-ACEI/ARB anti-hypertension drugs	Unadjusted comparisons	7
Tan et al. (8), China	100	11	NA	51%	DM: 28% CAD:(18%) CKD: (9%)	31	Patients with COVID-19 with hypertension that were taking non-ACEI/ARB anti-hypertension drugs.	Unadjusted comparisons	7
Tedeschi et al. (47), Italy	311	131	76 (67–83)	72%	CVD: 131 (42%) DM: 74 (24%) COPD: 49 (16%)	175	Patients with COVID-19 with hypertension that were taking non-ACEI/ARB anti-hypertension drugs.	Adjusted for age, gender, presence of CV comorbidities and COPD	8
Yan et al. (48), China	610	4	48.75 (±14.19)	51.10%	DM: 9.84% CVD: 2.62%	58	48,667 population-based controls from Zheijang, China with COVID-19 and hypertension that were taking non-ACEI/ARB anti-hypertension drugs.	Age, sex, BMI, and relevant comorbidities	8
Yang et al. (49), China	251	21	66.0 (60.0–73.0)	49%	DM: 55 (21.91%) CVD: 35 (13.94%) Pulmonary disease: 12 (4.78%) CKD: 4 (1.59%)	43	Patients with COVID-19 with hypertension that were taking non-ACEI/ARB anti-hypertension drugs.	Age, sex, BMI, complications (DM, pulmonary disease, hepatic disease, cardiopathy, neurological disease, immune diseases), other treatments (glucocorticoid, antiviral, antibiotic, immunoglobulin), and symptoms	8
Zeng et al. (50), China	274	21	NA	55%	DM: 42 (15%) CVD: 31 (11%)	28	Patients with COVID-19 with hypertension that were taking non-ACEI/ARB anti-hypertension drugs.	Age, sex, weight, BMI, comorbidities (obstructive pulmonary disease, CKD, CVD, DM, cerebrovascular disease, chronic liver disease, cancer), signs, and symptoms	7
Zhang et al. (51), China	522	NA	64 (56–69)	55.75%	DM: 126 (11.83%) CVD: 70 (13.41%) Pulmonary disease: 2 (0.38%) CKD: 18 (3.45%)	174	Patients with COVID-19 with hypertension that were taking non-ACEI/ARB anti-hypertension drugs.	Adjusted for age, gender, comorbidities (DM, coronary heart disease, cerebrovascular disease, and CKD), medication (antiviral drug and lipid lowering drug), symptoms, and signs.	9
Zhou et al. (52), China	36	7 (19.4%)	64.8 ± 10.1	53%	DM: 9 (25.0%) CAD:7 (19.4%)	15	Patients with COVID-19 with hypertension that were n taking non-ACEI/ARB anti-hypertension drugs.	age, sex, hospitalization time, time from onset to hospital admission	8
Zhou et al. (53), China	3,572	NA	66 (58–72)	51.10%	NA	989	Hospitalized patients with COVID-19 that were taking non-ACEI/ARB anti-hypertension drugs.	Adjustment for age, gender, disease severity, comorbidities, and CCB medication	7

*BMI, body mass index; CKD, chronic kidney disease; CVD, cardiovascular disease; DM, diabetes mellitus; IHD, ischaemic heart disease; NOS, Newcastle-Ottawa Scale*.

We carefully evaluated the quality of each study with the NOS. Thirty-eight studies (90.5%) had 7 points or more, and the remaining four studies had 6 points. Supplementary Table 2 shows the NOS scores of the included studies.

### Outcome Measures

#### Effects of ACEIs/ARBs on All-Cause Mortality in Patients With COVID-19

**Figure 2:**
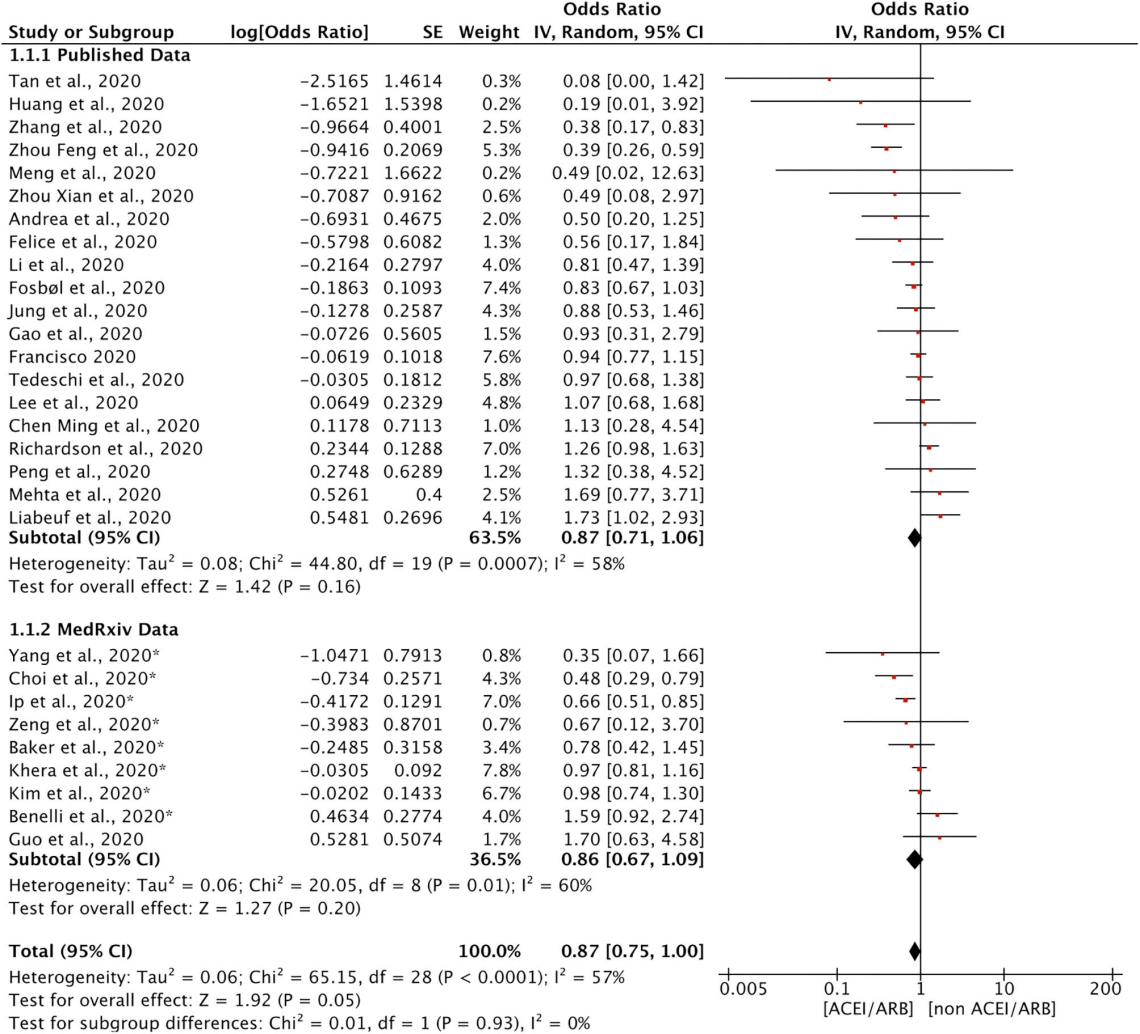
Forest plot showing the effects of ACEIs/ARBs on all-cause mortality in patients with COVID-19. SE, standard error; IV, inverse variance; df, degrees of freedom.

Twenty-nine studies discussed the relationship between the use of ACEIs/ARBs and all-cause mortality in patients with COVID-19 (Figure 2). The use of ACEIs/ARBs was not significantly associated with a reduction in the relative risk of all-cause mortality [OR = 0.87, 95% confidence interval (95% CI) = 0.75–1.00; *I*^2^ = 57%, *p* = 0.05]. The control groups generally included patients with COVID-19 who had taken other antihypertensive treatments. Most studies calculated the OR after adjusting for age, sex, and other factors to reduce the influence of confounding factors. To determine whether there was a difference between data from articles published in peer-reviewed journals and those posted on preprint servers, a subgroup analysis was conducted. There was no significant difference in the results for all-cause mortality between the two subgroups.

#### Effects of ACEIs and ARBs on the Severity of COVID-19

**Figure 3:**
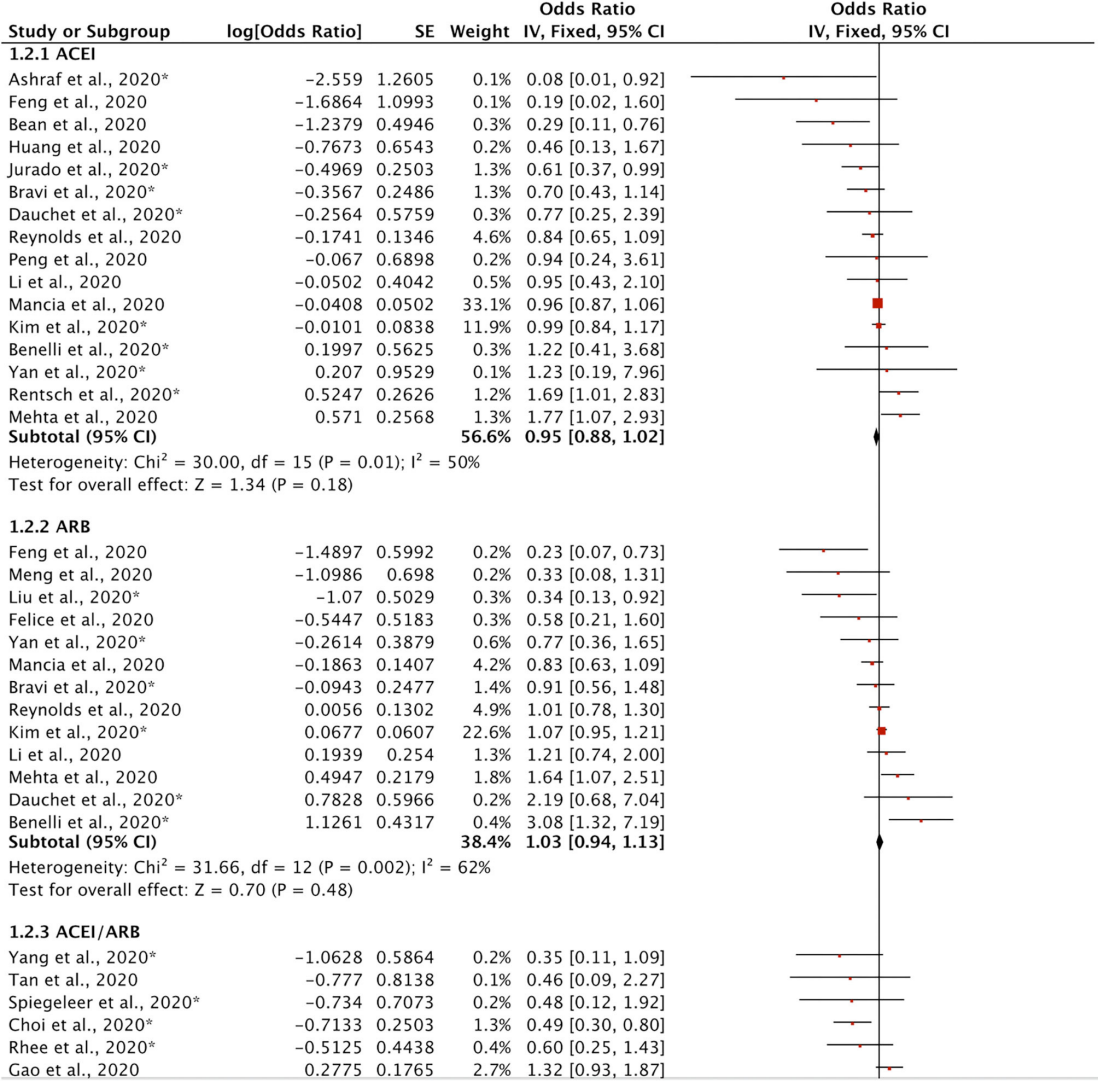
Forest plot showing the effects of ACEIs and ARBs on the severity of COVID-19. SE, standard error; IV, inverse variance; df, degrees of freedom.

Twenty-five retrospective studies evaluated the effects of ACEIs and ARBs on the severity of COVID-19 (Figure 3). We found no significant reduction in disease severity in the ACEI subgroup (OR = 0.95, 95% CI = 0.88–1.02, *I*^2^ = 50%, *p* = 0.18), in the ARB subgroup (OR = 1.03, 95% CI = 0.94–1.13, *I*^2^ = 62%, *p* = 0.48), or in the ACEI/ARB subgroup (OR = 0.83, 95% CI = 0.65–1.08, *I*^2^ = 67%, *p* = 0.16). Our meta-analysis demonstrated that there was no significant reduction in disease severity in patients taking ACEIs/ARBs (OR = 0.97, 95% CI = 0.92–1.03, *I*^2^ = 58%, *p* = 0.38). These findings indicate that ACEIs and ARBs might not have either a protective or adverse effect on disease severity.

#### Effect of ACEIs/ARBs on the Duration of Hospitalization for COVID-19 Treatment

**Figure 4:**
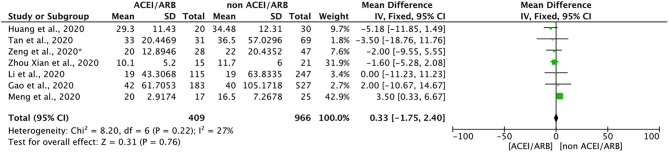
Forest plot showing the effects of ACEIs/ARBs on hospitalization duration in patients with COVID-19. SE, standard error; IV, inverse variance; df, degrees of freedom.

Seven included studies discussed the effects of ACEIs/ARBs on the duration of hospitalization required for the treatment of COVID-19. A meta-analysis of these studies showed that ACEIs/ARBs had no obvious effect on hospitalization duration (mean difference = 0.33, 95% CI = −1.75 to 2.40, *p* = 0.76). Because of the obvious limitation of the small number of included studies, we described these results qualitatively. In general, ACEIs/ARBs did not significantly shorten or prolong the hospitalization duration for patients with COVID-19 (Figure 4).

#### Sensitivity Analysis and Publication Bias

We performed a sensitivity analysis on the overall meta-analysis results. We performed sensitivity analyses for the effects of ACEIs/ARBs on the risks of mortality and severe disease by sequentially omitting one study at a time and investigating any changes in the findings. The results for all-cause mortality did not change significantly after excluding studies with low NOS scores, such as Li et al. (36). This finding indicated that the results were robust and reliable. Similarly, the pooled ORs for severe disease did not significantly change when we omitted studies one at a time.

We also evaluated publication bias with funnel plots. A visual inspection did not reveal any clear asymmetry (Supplementary Figures 1–3). Therefore, no significant publication bias was found among the included observational studies.

## Discussion

In the present meta-analysis, we found no significant association between the use of ACEIs/ARBs and the risks of mortality and severe disease in patients with COVID-19 after adjusting for baseline demographics and comorbidities (16, 36, 41, 44, 49, 57–59).

The concerns about the use of ACEIs or ARBs in patients with COVID-19 have mainly stemmed from arguments based on biologic plausibility, particularly the observation that ACEIs and ARBs have the potential to upregulate ACE2 receptors (which seem to be the mediators of the entry of SARS-CoV-2 into host cells) (60). However, it is also biologically plausible that ARBs may have beneficial effects in patients with COVID-19, although the findings have not been consistent across animal and human models (7). Therefore, ACE2 may act as a double-edged sword, depending on the phase of the disease. On the one hand, increased baseline ACE2 expression could potentially increase susceptibility of infection, making ACEI/ARB use a modifiable risk factor. On the other hand, once infected, the downregulation of ACE2 may be a hallmark of COVID-19 progression. Consequently, upregulation by preferentially blocking the RAS and replacing ACE2 in the acute respiratory syndrome phase may be beneficial. Our analysis supports that in the context of the current COVID-19 epidemic, the use of ACEIs/ARBs should not be restricted.

Several researchers found that the use of ACEIs/ARBs could worsen the prognosis of COVID-19 among patients with hypertension by promoting the expression of ACE2 (2–4, 61). These observational studies accounted for confounding factors, which is important because the factors that might indicate treatment with ACEIs or ARBs, such as comorbid cardiovascular conditions or diabetes, might also contribute to the development of severe COVID-19. We suspect that most of the patients taking RAS inhibitors had multiple comorbidities and cardiovascular risk factors, leading to a worse prognosis. Additionally, some of these studies' analyses were crude estimates that were not adjusted for confounding factors associated with hypertension, such as older age and cardiovascular disease. The adjustment of analyses is crucial for controlling for confounding factors, reducing bias, and increasing the reliability of the conclusions.

There have been three previous systematic reviews examining the effects of ACEI/ARB use in COVID-19 patients. Zhang et al. (51) found that ACEI/ARB exposure was not associated with a higher risk of severe disease or mortality. However, only 12 studies with unadjusted estimates were considered. Guo et al. (62) showed that ACEI/ARB use was associated with lower mortality in COVID-19 patients, although only six studies were included. Mackey et al. (63) conducted a narrative synthesis of 14 studies and concluded that there was no evidence of an association between ACEI/ARB use and severe COVID-19. In addition, Calderia et al. (64) and Barochiner and Martínez (65) drew similar conclusions, indicating that the use of ACEIs/ARBs does not increase the risk of severe COVID-19 or mortality; indeed, they suggested that the use of ACEIs/ARBs may have a protective effect. Our analysis included 48 studies and evaluated three outcomes. Additionally, to our knowledge, our review is the first to pool 35 adjusted effect estimates for mortality and severe COVID-19.

The results of the latest clinical trial, BRACE CORONA, have shown that the discontinuation of ACEIs/ARBs had no significant impact on the average survival duration or hospitalization duration (66). Currently, the European Society of Hypertension recommends continuing treatment with ACEIs/ARBs in patients with hypertension and COVID-19. These conclusions are consistent with those of our meta-analysis (67). We believe that the benefits of continuing treatment with ACEIs/ARBs outweigh the potential risks. Future well-designed randomized controlled trials and studies exploring the underlying mechanisms are needed to improve the level of evidence and determine whether the use of ACEIs/ARBs has an effect on the prognosis of patients with COVID-19.

## Limitation

First, although most of the available studies included in this meta-analyses reported adjusted estimates, some of the studies did not adjust the models, leading to an increased risk of bias in the pooled effect measures. Second, the majority of the included studies were observational in nature; thus, causality cannot be concluded because of the methodological limitations of this design. Third, heterogeneity was high in most of the evaluated outcomes. Possible reasons for the heterogeneity were the sample sizes, differences in outcome definitions, heterogeneous population, etc. Finally, the inconsistency of reporting the discontinuation of ACEIs or ARBs during hospitalization across studies could have influenced the pooled estimates.

## Conclusion

This meta-analysis suggested that ACEI/ARB use was not significantly associated with all-cause mortality in patients with hypertension who contracted COVID-19. In addition, ACEIs/ARBs had no significant effect on disease severity or the duration of hospitalization in COVID-19 patients with hypertension. This study provides additional evidence in favor of continuing antihypertension therapy after contracting COVID-19 unless the drugs cannot be tolerated because of hemodynamic instability.

## Data Availability Statement

The original contributions presented in the study are included in the article/Supplementary Material, further inquiries can be directed to the corresponding author.

## Author Contributions

Z-YA: analysis and interpretation of data, drafting the article, and reviewing and editing the article. X-CD, Z-YW, and Z-ZW: conception and design of the study, acquisition of data, and final approval of the version to be published. Z-YA and Y-RW: drafting the manuscript and checking the methodology and making the data curation. All authors: contributed to the article and approved the submitted version.

## Conflict of Interest

The authors declare that the research was conducted in the absence of any commercial or financial relationships that could be construed as a potential conflict of interest.
